# Nonlinear relationship between sleep midpoint and depression symptoms: a cross-sectional study of US adults

**DOI:** 10.1186/s12888-023-05130-y

**Published:** 2023-09-15

**Authors:** Jiahui Yin, Huayang Wang, Siyuan Li, Leiyong Zhao, Yanwei You, Jiguo Yang, Yuanxiang Liu

**Affiliations:** 1 College of Traditional Chinese Medicine, Shandong University of Traditional Chinese Medicine, Jinan, China; 2 Division of Mood Disorders, Shanghai Mental Health Center, Shanghai Jiao Tong University School of Medicine, Shanghai, China; 3 Department of Psychosomatic Medicine, Affliated Hospital of Shandong University of Traditional Chinese Medicine, Jinan, China; 4 Division of Sports Science & Physical Education, Tsinghua University, Beijing, China; 5 College of Acupuncture and Massage, Shandong University of Traditional Chinese Medicine, Jinan, China; 6 Department of Neurology, Shandong University of Traditional Chinese Medicine Affiliated Hospital, Jinan, China

**Keywords:** Depression symptoms, Biological rhythm, Sleep–wake cycle, Sleep midpoint

## Abstract

**Background:**

Despite the close relationship between sleep–wake cycles and depression symptoms, the relationship between sleep midpoint and depression symptoms in adults remains understudied.

**Methods:**

In this cross-sectional study, 18280 adults aged ≥ 18 years from the National Health and Nutrition Examination Survey (NHANES) 2015–2020 were analyzed. Covariates included age, sex, race/ethnicity, education level, marital status, family income, body mass index, smoking status, drinking status, physical activity, comorbid condition, sleep duration, and sleep disturbance were adjusted in multivariate regression models.

**Results:**

Weighted restricted cubic spline based on the complex sampling design of NHANES showed that in participants with a sleep midpoint from 2:18 AM to 6:30 AM, the prevalence of depression symptoms increased by 0.2 times (adjusted odds ratio [OR] = 1.20, 95% confidence interval [CI]: 1.08–1.33) per 1-h increment in sleep midpoint compared to the reference point of 2:18 AM. For participants with a sleep midpoint after 6:30 AM and before 2:18 AM the next day, the relationship between sleep midpoint and depression symptoms was not significant after adjusting for all covariates (adjusted OR = 1.01, 95% CI: 0.99–1.03).

**Conclusions:**

The findings indicate a significant nonlinear association between sleep midpoint and depression symptoms in a nationally representative sample of adults.

**Supplementary Information:**

The online version contains supplementary material available at 10.1186/s12888-023-05130-y.

## Background

In individuals with depression symptoms, disturbed biological rhythms are commonly observed [1–3]. In patients with major depressive disorder (MDD), a wide range of rhythms is disrupted, including sleep–wake cycles, hormone cycles (cortisol, melatonin), and the 24-h rhythmicity of circadian clock genes [4]. The disruption of biological rhythms can impair mood, behavior, and cognition, resulting in mood disorders [5]. Moreover, the disruption of biological rhythms is associated with severity of depression and suicidal ideation [6, 7].

Biological rhythms have been considered a potentially modifiable protective factor for depression symptoms [8]. It has been reported that the degree of antidepressant response to interventions is associated with the degree to which interventions alter circadian rhythms [9].

The sleep–wake cycle is a critical component of the circadian rhythm [10, 11]. Disturbance in the sleep–wake cycle is one plausible pathophysiological pathway of depressive disorders; patients with depressive disorders were found to benefit from interventions to sleep–wake disturbances [12]. Most of the effective treatments for depression symptoms, such as antidepressants (SSRIs, SNRIs, and melatonin receptor agonists), bright therapy, and exercise, can directly affect the sleep–wake cycle [13–15].

Previous studies of the relationship between the sleep–wake cycle and depression symptoms have focused on sleep duration [16], and prospective studies have found that short and long sleep durations are associated with a higher risk of depression symptoms [17]. Despite normal sleep duration, depression symptoms may still be affected by abnormal sleep midpoints (the midpoint between sleep time and wake time) [18].

Studies have found that a sleep midpoint outside 2:00–4:00 AM was associated with depressive symptoms in females [18, 19]. While Lin et al. found that the midpoint sleep time was positively associated with depressive symptom scores after 01:00 AM in older Taiwanese adults [20]. Daghlas et al. found that an 1-h earlier sleep midpoint was associated with a 23% lower risk of MDD through 2-sample mendelian randomization [21].

The results of studies on the relationship between sleep midpoint and depression symptoms are vague and heterogeneous. The National Health and Nutrition Examination Survey (NHANES) surveys a nationally representative sample of the US population through a complex, stratified, multistage probability cluster sampling design. Therefore, we conducted a cross-sectional study using data from NHANES to explore the association between sleep midpoint and depression symptoms in US adults.

## Materials and methods

### Participants

The participants in this study were obtained from the NHANES, a major program conducted by the Centers for Disease Control and Prevention (CDC) to assess the health and nutritional status of adults and children in the US. NHANES is a cross-sectional survey, conducted by the National Center for Health Statistics (NCHS), that includes a nationally representative sample of noninstitutionalized, resident US civilians of all ages. The NHANES comprises demographic, socioeconomic, health-related, and medical information. The National Center for Health Statistics Research Ethics Review Board authorized the NHANES study protocols. Since 1999, the NHANES has been conducted every two years and has used a stratified, multistage probability sampling approach. Further information regarding the NHANES is available on the CDC website [22]. The data of this study were collected during the NHANES survey cycle from 2015 to 2020.

Participants in our study were screened according to the following exclusion criteria: 1) Younger than 18 years old; 2) incomplete Patient Health Questionnaire-9 (PHQ-9); and 3) missing information on sleep midpoint.

### Exposure: sleep midpoint

The sleep midpoint was calculated based on self-reported waking and initial sleep times; the midpoint between going to sleep and the time of awakening was defined as the sleep midpoint. Information on waking and initial sleep times was acquired based on answers to the following questions: “What time do you usually go to sleep on weekdays or workdays?”; “What time do you usually wake up on weekdays or workdays?” This access method is very commonly used when studying the sleep midpoint [19, 20, 23].

### Outcome: depression symptoms

The PHQ-9, which is a questionnaire based on self-reporting by patients, was used to assess depression symptoms. It contains nine items (depressed mood, appetite problems, fatigue, sleep difficulties, psychomotor retardation or agitation, concentration problems, lack of interest, feelings of worthlessness, and suicidal ideation) that assess the frequency of depressive symptoms and is well accepted as an accurate and reliable method for depression symptoms screening [24–27]. The total PHQ-9 score varies from 0 to 27; a score of 10 or greater was defined as indicative of depression symptoms.

### Covariates

Adjustment covariates were chosen based on previous literature [28–32]. Age, body mass index (BMI), and sleep duration were continuous variables. BMI was measured as weight (kg) divided by height (m) squared. Sleep duration was determined by answers to the question, “How much sleep do you usually get at night on weekdays or workdays?” Sex (male/female), race/ethnicity (non-Hispanic white, Mexican American, non-Hispanic black, other Hispanic or other race/multiple races), education level (< high school/completed high school/ > high school), marital status (married/living with a partner or never married/widowed /divorced/separated), physical activity (inactive/moderate/vigorous/both moderate and vigorous), family income, smoking status, drinking status, and comorbid condition were used as categorical variables. Family income was categorized into the following 3 levels based on the family poverty income ratio: low income (≤ 1.3), medium income (> 1.3 to 3.5), and high income (> 3.5). Drinking status was categorized as never (had < 12 drinks in a lifetime), former (had ≥ 12 drinks in 1 year and did not drink last year, or did not drink last year but drank ≥ 12 drinks in a lifetime), current light/moderate drinker (≤ 1 drink per day for females or ≤ 2 drinks per day for males on average over the past year), or current heavier drinker (> 1 drink per day for females or > 2 drinks per day for males on average over the past year). Smoking status was categorized into “never smoker,” “former smoker,” or “current smoker.” Participants were defined as a "never smoker" if they answered "No" to "Ever smoked a cigarette even one time" or "Smoked at least 100 cigarettes in life". If participants answered "Yes" to "Ever smoked a cigarette even one time," then they were excluded from the category of "never smoker." Participants were defined as a "former smoker" if they were not currently smoking but answered "Yes" to "Ever smoked a cigarette even one time" or "Smoked at least 100 cigarettes in life." Participants were defined as a "current smoker" if they answered "Every day" or "Some days" to "Do you now smoke cigarettes?". Physical activity was assessed as vigorous physical activity (high-intensity activities, fitness, and sports such as running or basketball) and moderate physical activity (e.g., brisk walking, swimming, bicycling at a regular pace), reported by participants. Participants were defined as having the comorbid condition if they reported at least one of the following medical conditions: diabetes, kidney failure, kidney stones, heart failure, stroke, hepatopathy, rheumatoid arthritis, and cancer. Because of the non-orthogonal relationship between sleep midpoint and depression symptoms, we adjusted the sleep item of the PHQ-9 questionnaire as a covariate. In this item, participants reported the frequency of sleep disturbance (Not at all/Several days/More than half the days/Nearly every day).

### Statistical analyses

**Fig. 1:**
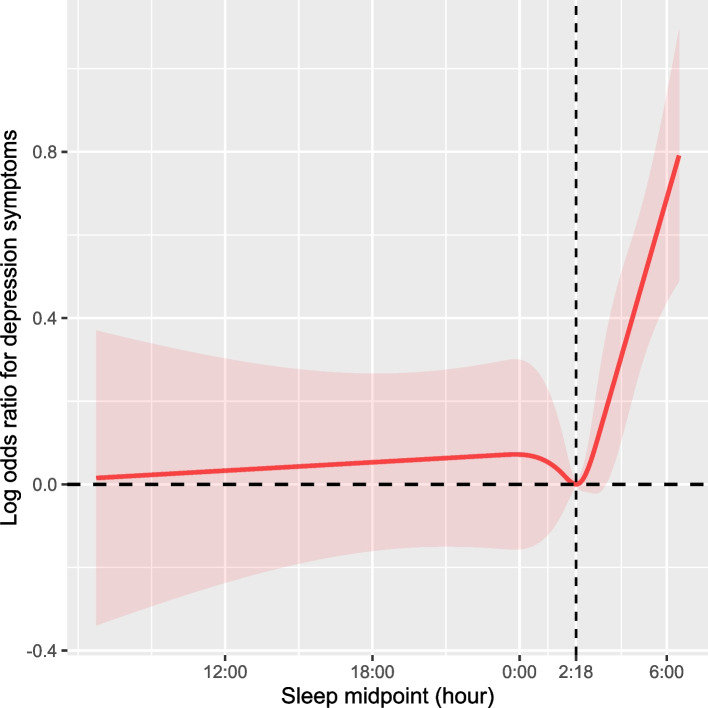
Weighted restricted cubic spline of the association between sleep midpoint and depression symptoms. Shaded areas are 95% confidence intervals. The model is adjusted for age, sex, race/ethnicity, education level, marital status and family income, body mass index, smoking status, drinking status, physical activity, comorbid condition, sleep duration and sleep disturbance (Model 3)

All analyses accounted for the NHANES complex sampling design to derive nationally representative estimates [33]. The characteristics of participants are described as means (95% CIs) for continuous variables and percentage frequencies (95% CIs) for categorical variables. Continuous data were compared using analysis of variance, and the χ^2^ test compared categorical data. The nonlinear relationship between sleep midpoint and depression symptoms was tested using restricted cubic splines (RCS) (Fig. 1). A *P* value for nonlinear < 0.05 indicates a nonlinear relationship [34–37]. Multivariable logistic regression analysis was performed to evaluate the associations between sleep midpoint and depression symptoms. We used three levels of adjustment. In Model 1, age, sex, and race/ethnicity were chosen as covariates because they are basic demographic information and have been shown to be associated with sleep behaviors and depression symptoms. Model 2 was adjusted for the variables in Model 1 plus education level, marital status, physical activity, and family income, because these are sociological factors that are commonly adjusted for when exploring depression symptoms and sleep behaviors. These variables can influence an individual's lifestyle, social support, and access to healthcare resources, which could affect their sleep quality and depressive conditions. In addition to variables in Models 1 and 2, Model 3 was adjusted for BMI, smoking status, drinking status, physical activity, comorbid condition, sleep duration, and sleep disturbance. These variables were included because lifestyle habits and health status are known to impact both sleep quality and depression symptoms. Covariates that showed a nonlinear relationship with the outcome in the univariate analysis were adjusted as RCSs. Imputation of missing data was conducted using the missForest R package, which is a random forest-based technique that is highly computationally efficient for high-dimensional data consisting of both categorical and continuous predictors [38]. Missing covariates are listed in Table S1.

We further examined the threshold effect of sleep midpoint on depression symptoms. A likelihood ratio test comparing the one-line model with a piecewise regression model was used to determine whether a threshold exists. The threshold level of the sleep midpoint was determined using a recurrence method, including selecting the turning point along a predefined interval and choosing the turning point that yielded the maximum likelihood model. To further assess the robustness of the association between sleep midpoint and depression symptoms, we conducted stratified RCS.

All analyses were performed using R (R Foundation for Statistical Computing, Vienna, Austria). A two-sided *p*-value < 0.05 was considered statistically significant.

## Results

### Participant characteristics

A total of 18,280 participants were included in this study. A flow chart of the included/excluded participants is shown in Figure S1. Table 1 shows the characteristics of the participants (Q1: > 6:30 PM to ≤ 1:30 AM the next day; Q2: > 1:30 AM to ≤ 2:30 AM; Q3: > 2:30 AM to < 3:25 AM; Q4: ≥ 3:25 AM to ≤ 6:30 AM). Participants in Group 4 had the highest prevalence of depressive symptoms and more frequent sleep disturbances. Compared to participants in other groups, those in Group 4 had the lowest average age and BMI, and the longest sleep duration. Additionally, Group 4 had the highest proportion of participants who were physically inactive and had low income. Furthermore, the lowest proportion of participants who were married or living with a partner among all participants was in Group 4 (all *P* < 0.05).

**Table 1:** Characteristics of participants in the NHANES 2015–2020 Cycles (*n* = 18,280)

Characteristic^a^	Overall	Sleep midpoint groups				*p*-value
		Group 1	Group 2	Group 3	Group 4	
		> 6:30 PM to ≤1:30 AM the next day	> 1:30 AM to ≤2:30 AM	> 2:30 AM to < 3:25 AM	≥3:25 AM to ≤6:30 AM	
	(*N* = 18,280)	(*n* = 4745)	(*n* = 5645)	(*n* = 3329)	(*n* = 4561)	
Age, mean (95% CI), y	47.38 (46.69–48.08)	48.10 (47.24–48.95)	48.12 (47.40–48.83)	48.82 (47.53–50.11)	44.42 (43.30–45.53)	< 0.001
Sex						< 0.001
Female	9326 (51.41) [48.64–54.19]	2203 (46.68) [44.57–48.79]	3009 (54.16) [52.06–56.26]	1763 (52.59) [50.01–55.16]	2351(51.78) [49.77–53.79]	
Male	8954 (48.59) [46.08–51.10]	2542 (53.32) [51.21–55.43]	2636 (45.84) [43.74–47.94]	1566 (47.41) [44.84–49.99]	2210 (48.22) [46.21–50.23]	
Educational level						< 0.001
< High school	3514 (11.69) [10.55–12.83]	1132 (14.11) [11.99–16.24]	1111 (11.68) [10.18–13.17]	517 (9.03) [7.82–10.24]	754 (11.79) [10.42–13.15]	
Completed high school	4316 (25.29) [23.49–27.08]	1310 (30.94) [28.57–33.32]	1223 (22.86) [20.94–24.77]	667 (19.93) [17.69–22.17]	1116 (28.10) [25.58–30.62]	
> High school	10,051 (61.97) [57.70–66.24]	2256 (54.94) [52.43–57.45]	3235 (65.47) [62.64–68.30]	2071 (71.03) [68.37–73.70]	2489 (60.11) [56.89–63.34]	
Race/ethnicity						< 0.001
Non-Hispanic White	6379 (63.78) [58.10–69.46]	1517 (62.17) [57.76–66.59]	1979 (64.92) [60.82–69.03]	1264 (67.27) [64.22–70.32]	1619 (61.05) [57.79–64.31]	
Non-Hispanic Black	4370 (11.05) [9.28–12.81]	1340 (13.03) [10.40–15.65]	1292 (10.55) [8.42–12.67]	671 (8.55) [6.92–10.18]	1067 (11.66) [9.97–13.34]	
Mexican American	2595 (8.90) [6.99–10.81]	872 (11.55) [8.44–14.65]	808 (8.45) [6.62–10.27]	421 (7.73) [6.02- 9.43]	494 (7.62) [5.90- 9.34]	
Other Hispanic	1940 (6.80) [5.75- 7.86]	456 (6.14) [5.19–7.08]	690 (7.81) [6.35–9.27]	336 (5.70) [4.48–6.91]	458 (7.06) [5.73–8.39]	
Other race/multiple races	2996 (9.47) [8.28–10.66]	560 (7.12) [6.07- 8.16]	876 (8.27) [6.85- 9.70]	637 (10.76) [9.06–12.45]	923 (12.62) [10.75–14.49]	
Marital status						< 0.001
Married/Living with partner	10,275 (61.39) [57.75–65.03]	2728 (62.72) [60.38–65.06]	3469 (68.88) [66.87–70.88]	1927 (65.52) [63.15–67.90]	2151 (54.04) [51.34–56.74]	
Never married/Widowed /Divorced/Separated	7120 (35.59) [33.47–37.71]	1913 (37.28) [34.94–39.62]	2002 (31.12) [29.12–33.13]	1230 (34.48) [32.10–36.85]	1975 (45.96) [43.26–48.66]	
Alcohol status						< 0.001
Never drinking	2321 (9.35) [8.47–10.22]	577 (10.07) [8.64–11.50]	795 (11.39) [10.12–12.66]	437 (10.18) [8.41–11.96]	512 (9.96) [8.78–11.15]	
Former drinker	817 (4.33) [3.80- 4.86]	239 (6.24) [5.37–7.11]	246 (3.80) [3.27–4.33]	149 (4.68) [3.36–6.00]	183 (5.01) [3.79–6.24]	
Current light/moderate drinker	9205 (55.08) [51.62–58.53]	2194 (57.03) [54.83–59.23]	2905 (63.61) [61.06–66.16]	1779 (65.54) [62.96–68.12]	2327 (61.58) [58.30–64.85]	
Current heavier drinker	3348 (20.28) [18.79–21.78]	972 (26.67) [24.34–28.99]	958 (21.20) [18.95–23.44]	530 (19.60) [17.64–21.55]	888 (23.44) [21.05–25.84]	
Smoking status						< 0.001
Never smoker	10,800 (48.90) [46.22–51.57]	2621 (46.26) [43.59–48.94]	3458 (56.81) [54.73–58.89]	2035 (57.49) [54.42–60.56]	2686 (54.11) [51.40–56.82]	
Former smoker	4204 (25.13) [23.13–27.13]	1157 (31.07) [28.87–33.27]	1282 (26.72) [24.63–28.81]	769 (26.80) [24.42–29.18]	996 (25.56) [23.07–28.06]	
Current smoker	3268 (17.15) [15.90–18.39]	965 (22.67) [20.55–24.79]	901 (16.47) [14.67–18.28]	523 (15.71) [13.88–17.54]	879 (20.32) [18.62–22.03]	
Physical activity						< 0.001
Inactive	9353 (44.53) [41.95–47.12]	2575 (47.58) [45.06–50.09]	2846 (42.46) [39.56–45.36]	1574 (39.69) [37.06–42.32]	2358 (48.10) [45.04–51.17]	
Moderate	4233 (25.93) [23.99–27.88]	1054 (25.45) [22.76–28.14]	1350 (26.27) [24.35–28.18]	859 (30.25) [27.73–32.78]	970 (22.42) [20.31–24.52]	
Vigorous	1410 (8.18) [ 7.38- 8.98]	317 (8.13) [7.13–9.12]	414 (7.90) [6.69–9.10]	292 (8.27) [6.82–9.71]	387 (8.56) [7.51–9.61]	
Both moderate and vigorous	3284 (21.35) [19.35–23.35]	799 (18.84) [16.79–20.90]	1035 (23.38) [20.78–25.98]	604 (21.79) [18.79–24.79]	846 (20.92) [18.45–23.39]	
BMI, mean (95% CI), kg/m^2^	29.61 (29.33–29.89)	30.04 (29.73–30.35)	29.53 (29.16–29.90)	29.43 (29.01–29.84)	29.41 (28.93–29.89)	0.02
Family income^b^						< 0.001
Low income	4801 (18.11) [16.97–19.24]	1278 (21.11) [19.38–22.84]	1410 (16.66) [14.69–18.64]	747 (15.90) [13.80–18.01]	1366 (26.84) [24.34–29.34]	
Medium income	6391 (32.43) [30.15–34.71]	1699 (37.69) [34.93–40.45]	1887 (34.13) [31.62–36.65]	1150 (33.58) [30.87–36.29]	1655 (38.05) [34.65–41.45]	
High income	4904 (39.97) [36.49–43.46]	1192 (41.20) [37.81–44.60]	1682 (49.20) [45.76–52.65]	1030 (50.52) [47.22–53.82]	1000 (35.12) [31.88–38.35]	
Sleep duration, mean (95% CI), h	7.64 (7.60–7.67)	7.35 (7.28–7.42)	7.59 (7.53–7.64)	7.73 (7.67–7.79)	7.93 (7.87–7.98)	
Sleep disturbance						< 0.001
Not at all	11,214 (60.31) [57.11,63.50]	2989 (61.15) [59.00,63.29]	3717 (66.29) [64.61,67.97]	2115 (62.50) [59.84,65.17]	2393 (49.33) [47.41,51.26]	
Several days	4172 (24.04) [22.64,25.43]	997 (21.92) [20.55,23.29]	1213 (21.67) [20.06,23.27]	754 (24.38) [22.30,26.46]	1208 (29.31) [27.57,31.05]	
More than half the days	1323 (7.90) [7.16, 8.64]	312 (7.75) [6.49, 9.01]	349 (6.80) [5.77, 7.84]	234 (7.46) [6.26, 8.65]	428 (9.93) [8.60,11.26]	
Nearly every day	1571 (7.76) [7.08, 8.44]	447 (9.18) [7.98,10.38]	366 (5.24) [4.49, 5.99]	226 (5.67) [4.67, 6.66]	532 (11.43) [10.20,12.66]	
Comorbid condition						< 0.001
No	9843 (56.76) [53.43–60.09]	2399 (52.94) [50.35–55.53]	3145 (59.62) [57.73–61.52]	1778 (56.38) [53.44–59.33]	2521 (57.44) [54.89–59.99]	
Yes	8417 (43.17) [40.58–45.76]	2342 (47.06) [44.47–49.65]	2500 (40.38) [38.48–42.27]	1544 (43.62) [40.67–46.56]	2031 (42.56) [40.01–45.11]	
PHQ-9 score, mean (95% CI)^c^	3.13 (3.04,3.22)	3.13 (2.96,3.30)	2.65 (2.51,2.79)	2.79 (2.60,2.99)	4.06 (3.90,4.22)	< 0.001
Depression symptoms						< 0.001
No	16,680 (91.89) [87.42–96.37]	4308 (91.70) [90.50–92.89]	5240 (94.04) [93.30–94.78]	3134 (94.32) [92.77–95.86]	3998 (87.15) [85.92–88.39]	
Yes	1600 (8.11) [7.38- 8.83]	437 (8.30) [7.11- 9.50]	405 (5.96) [5.22- 6.70]	195 (5.68) [4.14- 7.23]	563 (12.85) [11.61–14.08]	

*Abbreviations*: *NHANES* National Health and Nutrition Examination Survey, *CI* Confidence interval, *BMI* Body mass index, *SE* Standard error, *Q* Quantile

^a^Data are presented as unweighted number (weighted percentage) [95% CI] unless otherwise indicated

^b^Categorized into the following 3 levels based on the family poverty income ratio: low income (< = 1.3), medium income (> 1.3 to 3.5), and high income (> 3.5)

^c^PHQ-9 scores range from 0 to 27, with a score greater than 9 used to indicate depression symptoms in this study

A comparison of characteristics between participants included and excluded (excluded for the absence of sleep midpoint data or incomplete PHQ-9) from the analysis is shown in Table S2. In addition, we used univariate analysis to observe the association between covariates and depression symptoms (Table S3).

### Association of sleep midpoint and depression symptoms

As shown in Fig. 1, RCSs suggested that the relationship between sleep midpoint and depression symptoms was nonlinear. Table 2 shows the results of the multivariable logistic regression analysis. Sleep midpoint was converted into a categorical variable; after adjusting for all covariates, compared with participants in Group 2, participants in Group 4 (odds ratio [OR] = 1.25, 95% confidence interval [CI]: 1.02–1.53), had an increased prevalence of depression symptoms; participants in Group 1 (OR = 0.93, 95%CI: 0.71–1.22) and Group 3 (OR = 0.83, 95%CI: 0.54–1.28) had no significant difference in the prevalence of depression symptoms.

**Table 2:** Associations of sleep midpoint and depression symptoms (*n* = 18,280)

Variables	Crude model^a^		Model 1^b^		Model 2^c^		Model 3^d^	
	OR (95% CI)	*p*-value	OR (95% CI)	*p*-value	OR (95% CI)	*p*-value	OR (95% CI)	*p*-value
per 1 h increase	0.99 (0.97, 1.01)	0.426	0.99 (0.96, 1.01)	0.313	0.99 (0.97, 1.02)	0.607	1.02 (1.00, 1.04)	0.018
Group 1 (> 6:30 AM to ≤ 1:30 AM the next day)	1.43 (1.16, 1.76)	0.001	1.50 (1.22, 1.84)	< 0.001	1.36 (1.08, 1.70)	0.009	0.93 (0.71, 1.22)	0.575
Group 2 (> 1:30 AM to ≤ 2:30 AM)	Reference (1)		Reference (1)		Reference (1)		Reference (1)	
Group 3 (> 2:30 AM to < 3:25 AM)	0.95 (0.69, 1.31)	0.752	0.96 (0.70, 1.32)	0.806	0.95 (0.68, 1.32)	0.738	0.83 (0.54, 1.28)	0.39
Group 4 (≥ 3:25 AM to ≤ 6:30 AM)	2.32 (1.96, 2.76)	< 0.001	2.34 (1.97, 2.78)	< 0.001	1.98 (1.65, 2.39)	< 0.001	1.25 (1.02, 1.53)	0.035

*Abbreviations*: *CI* Confidence interval, *OR* Odds ratio, *RCS* Restricted cubic spline

Covariates that showed a nonlinear relationship with the outcome in the univariate analysis were adjusted as RCSs

^a^Crude Model: Unadjusted

^b^Model 1: Adjust for age, sex, and race/ethnicity

^c^Model 2: Adjust for the variables in Model 1 plus education level, marital status and family income

^d^Model 3: Adjust for the variables in Model 2 plus body mass index (RCS), smoking status, drinking status, physical activity, comorbid condition, sleep duration (RCS), and sleep disturbance

According to the threshold effect analysis, after adjusting for all covariates, in participants with a sleep midpoint from 2:18 AM to 6:30 AM, the prevalence of depression symptoms increased by 0.2 times (unadjusted OR = 1.55, 95% CI: 1.43–1.67; adjusted OR = 1.20, 95% CI: 1.08–1.33) per 1-h increment in sleep midpoint compared to the reference point of 2:18 AM. For participants with a sleep midpoint after 6:30 AM and before 2:18 AM the next day, the relationship between sleep midpoint and depression symptoms was not significant after adjusting for all covariates (unadjusted OR = 0.94, 95% CI: 0.92–0.95; adjusted OR = 1.01, 95% CI: 0.99–1.03) (Table 3). Using imputation and setting dummy variables as methods for handling missing covariates gave consistent results in multivariable logistic regressions and threshold effect analyses (Tables 2, 3, and S4).

**Table 3:** Threshold effect analysis of sleep midpoint on depression symptoms using two-piecewise logistic regression mode (*n* = 18,280)

Depression symptoms	Crude model^a^		Adjusted model^b^	
	OR (95% CI)	*p*-value	OR (95% CI)	*p*-value
Sleep midpoint after 6:30 AM and before 2:18 AM the next day	0.94 (0.92, 0.95)	< 0.001	1.01 (0.99, 1.03)	0.291
Sleep midpoint from 2:18 AM to 6:30 AM	1.55 (1.43, 1.67)	< 0.001	1.20 (1.08, 1.33)	0.002
*P* value for nonlinear	< 0.001		< 0.001	

*Abbreviations*: *CI* Confidence interval, *OR* Odds ratio, *RCS* Restricted cubic spline

Covariates that showed a nonlinear relationship with the outcome in the univariate analysis were adjusted as RCSs

^a^Crude Model: Unadjusted

^b^Adjusted model: Adjusted for all covariates in model 3: age, sex, race/ethnicity, education level, marital status, family income, body mass index (RCS), smoking status, drinking status, physical activity, comorbid condition, sleep duration (RCS) and sleep disturbance

**Fig. 2:**
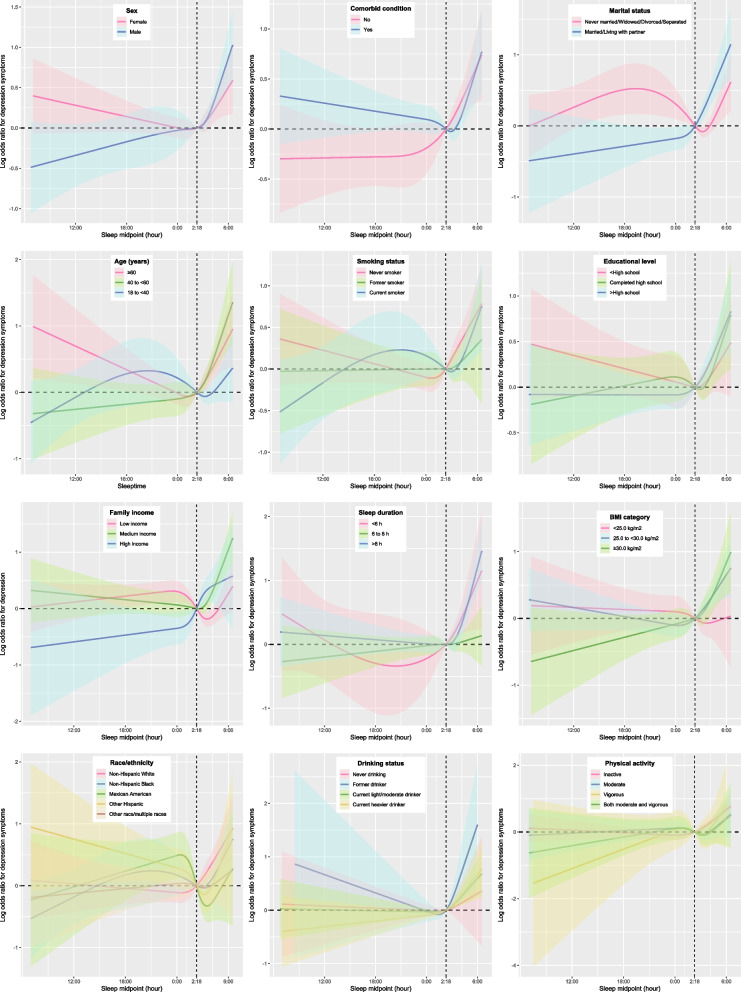
Weighted stratified restricted cubic spline of the association between sleep midpoint and depression symptoms. Shaded areas are 95% confidence intervals. Analyses were adjusted for covariates: age, sex, race/ethnicity, education level, marital status and family income, body mass index, smoking status, drinking status, physical activity, comorbid condition, sleep duration and sleep disturbance (Model 3). Stratified variables were not adjusted in the corresponding model

Figure 2 illustrates the relationship between sleep midpoint and depression symptoms in different subgroups. In this study, depression symptoms were assessed using the PHQ-9. To examine the robustness of the results, we also analyzed the PHQ-9 scores as a continuous variable. We plotted the RCS of the sleep midpoint and the PHQ-9 score (with the sleep subitem removed) in sensitivity analyses (Fig. S2). RCS and threshold effect analysis showed that the relationship between sleep midpoint and PHQ-9 score was still nonlinear (Table S5).

## Discussion

In this study, we examined the relationship between sleep midpoint and depression symptoms in adults in a nationally representative sample of the US population. A nonlinear relationship was found between sleep midpoint and depression symptoms. The depression symptoms prevalence increased by 0.2 times with each hour increase in sleep midpoint from 2:18 AM to 6:30 AM. The relationship between sleep midpoint and depression symptoms was not significant for participants with a sleep midpoint after 6:30 AM and before 2:18 AM the next day. For the first time, weighted RCS based on the complex sampling design was plotted, which more graphically showed the relationship between sleep midpoint and depression symptoms across diverse population subgroups. We found that for participants whose sleep midpoint was later than the inflection point, there was an increasing trend in the prevalence of depression symptoms with delayed sleep midpoint.

Most previous studies on the relationship between sleep midpoint and depression symptoms were conducted in the elderly population, but there has been a lack of research in adults. As far as we know, this is the study with the largest sample size (*n* = 18280) to date exploring the relationship between sleep midpoint and depression symptoms in a nationally representative sample. Daghlas et al. found that an earlier 1-h sleep midpoint was associated with a 23% lower risk of MDD. Still, they did not test for nonlinear associations because of the limitations inherent in the two-sample Mendelian randomization study [21]. Lin et al. found a significant positive correlation between the sleep midpoint and depressive symptom scores after 01:00 AM in the older adult population [20]. Studies have also found that sleep midpoints outside of 2:00–4:00 AM are associated with depressive symptoms in elderly women [18, 19]. In addition, previous studies on shift workers have found that they have a late sleep midpoint and more depression symptoms [39, 40]. Our study found a nonlinear relationship between sleep midpoint and depression symptoms in US Adults and the exact inflection point (2:18 AM) when the insignificant correlation between sleep midpoint and depression symptoms becomes significantly positive.

The potential mechanisms of the relationship between sleep midpoint and depression symptoms include genetics, neurohumoral systems, molecular expressions, and behavioral levels. Alterations in genes such as *CLOCK* [41], *5-HTTLPR* [42], *PER* [43], *NPAS2* [44], and *CRY*s [45] distort sleep and are additionally associated with the pathophysiology of the sleep–wake cycle. In addition, impaired HPA axis function and impaired melatonergic systems are thought to affect the sleep–wake cycle and contribute to depression symptoms [46, 47].

This study may guide research on circadian rhythm and depression symptoms. Exercise [48], light therapy [49], melatonin, and its analogs [47] can regulate sleep–wake rhythm and have antidepressant effects. It is necessary to conduct prospective studies to explore the relationship between sleep midpoint and depression symptoms and the potential effect of sleep midpoint interventions on depression symptoms.

This study has several strengths. Analyses used a large probability-based sample and sampling weights; therefore, the conclusions of this study can be generalized across adults in the US. Furthermore, we plotted weighted RCS based on the complex sampling design, which more graphically showed the relationship between sleep midpoint and depression symptoms across diverse population subgroups.

The study also has some limitations. First, cross-sectional studies cannot determine causality, and further prospective studies are needed to explore the effects of changing sleep midpoints on depression symptoms. Second, this study focused on patients with depression symptoms, and further research is required to generalize the findings to patients with MDD. Additionally, our assessment of the sleep midpoint was based on self-reported information, which may produce measurement errors. However, investigations have shown moderately good correlations between subjective estimates and sleep diaries, actigraphy, or polysomnography [50]. Sleep midpoint measurements based on self-reporting is also more affordable and accessible, and primary health care assessments rely on patient self-reporting. Finally, shift workers have a specific sleep schedule, but they could not be excluded or analysed separately in this study. This is because information on shift work was not collected in the NHANES survey. Future studies are needed to investigate the ralationship between sleep midpoint and depression symptoms in shift workers.

## Conclusions

Based on a multiethnic sample, this cross-sectional study found a nonlinear relationship between sleep midpoint and depression symptoms. The results support further research into the effectiveness of sleep-timing interventions for preventing and treating depression symptoms in randomized clinical trials.

### Supplementary Information

**Additional file 1.**

## Data Availability

The data used in this study are available on the National Health and Nutrition Examination Survey website: https://wwwn.cdc.gov/nchs/nhanes/ContinuousNhanes/Default.aspx.

## Abbreviations

BMI: Body mass index
CDC: Centers for Disease Control and Prevention
CI: Confidence interval
MDD: Major depressive disorder
NHANES: National Health and Nutrition Examination Survey
OR: Odds ratio
PHQ-9: Patient Health Questionnaire-9
RCS: Restricted cubic spline
SD: Standard deviation
