# Supplementary material for: Nonlinear relationship between sleep midpoint and depression symptoms: a cross-sectional study of US adults
Source: BMC Psychiatry. 2023 Sep 15;23:671. doi: 10.1186/s12888-023-05130-y (PMC10503124; doi:10.1186/s12888-023-05130-y)
Supplement: Supplementary file 1 — Additional file 1. [file 12888_2023_5130_MOESM1_ESM.docx]

**Supplementary material**


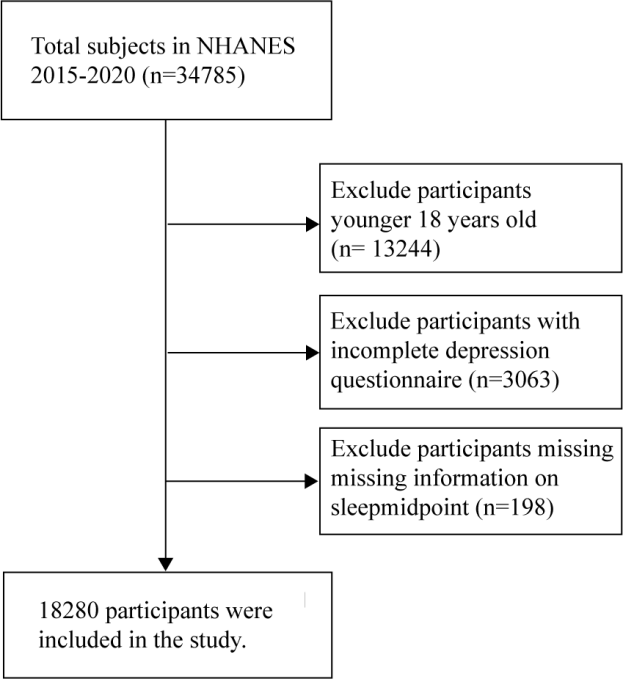


Figure S1. Flowchart for inclusion of study participants.

Table S1. Missing covariates (n = 18280).

| Variable | Number of patients (% missing) |
| --- | --- |
| Age | 0 (0%) |
| Sex | 0 (0%) |
| Race/ethnicity | 0 (0%) |
| Educational level | 399 (2.18%) |
| Marital status | 885 (4.84%) |
| Drinking status | 2589 (14.16%) |
| Smoking status | 1318 (7.21%) |
| Physical activity | 0 (0%) |
| Body mass index | 204 (1.12%) |
| Family income | 2184 (11.95%) |
| Comorbid condition | 20 (0.11%) |
| Sleep duration | 1 (0.01%) |
| Sleep disturbance | 0 (0%) |

Table S2. Characteristics of the included and excluded participants

| Characteristic | Excluded | Included | p-value |  |
| --- | --- | --- | --- | --- |
|  |  |  |  |  |
|  |  |  |  |  |
| Sample size | 3261 | 18280 |  |  |
| Male, n (%) | 1491 (45.7) | 8954 (49.0) | 0.001 |  |
| Age, y, mean (SD) | 50.72 (19.77) | 49.00 (18.42) | <0.001 |  |
| Educational level, n (%) |  |  | <0.001 |  |
| <High school | 893 (28.2) | 3514 (19.7) |  |  |
| Completed high school | 737 (23.3) | 4316 (24.1) |  |  |
| >High school | 1536 (48.5) | 10051 (56.2) |  |  |
| Race/ethnicity, n (%) |  |  | <0.001 |  |
| Non-Hispanic White | 937 (28.7) | 6379 (34.9) |  |  |
| Non-Hispanic Black | 793 (24.3) | 4370 (23.9) |  |  |
| Mexican American | 392 (12.0) | 2595 (14.2) |  |  |
| Other Hispanic | 392 (12.0) | 1940 (10.6) |  |  |
| Other race/multiple races | 747 (22.9) | 2996 (16.4) |  |  |
| BMI, kg/m^2^, mean (SD) | 28.89 (7.38) | 29.77 (7.42) | <0.001 |  |
| Marital status, n (%) |  |  | <0.001 |  |
| Married/Living with partner | 1697 (54.6) | 10275 (59.1) |  |  |
| Never married/Widowed /Divorced/Separated | 1409 (45.4) | 7120 (40.9) |  |  |
| Drinking status, n (%) |  |  | <0.001 |  |
| Never drinking | 110 (36.2) | 2321 (14.8) |  |  |
| Former drinker | 28 (9.2) | 817 (5.2) |  |  |
| Current light/moderate drinker | 125 (41.1) | 9205 (58.7) |  |  |
| Current heavier drinker | 41 (13.5) | 3348 (21.3) |  |  |
| Smoking status, n (%) |  |  | <0.001 |  |
| Never smoker | 1829 (60.4) | 9490 (55.9) |  |  |
| Former smoker | 661 (21.8) | 4204 (24.8) |  |  |
| Current smoker | 537 (17.7) | 3268 (19.3) |  |  |
| Physical activity, n (%) |  |  |  |  |
| Inactive | 1896 (58.1) | 9353 (51.2) | <0.001 |  |
| Moderate | 644 (19.7) | 4233 (23.2) |  |  |
| Vigorous | 227 (7.0) | 1410 (7.7) |  |  |
| Both moderate and vigorous | 494 (15.1) | 3284 (18.0) |  |  |
| Comorbid condition, n (%) | 1370 (42.2) | 8417 (46.1) | <0.001 |  |
| Family income, n (%) |  |  |  |  |
| Low income | 826 (33.7) | 4801 (29.8) | <0.001 |  |
| Medium income | 963 (39.3) | 6391 (39.7) |  |  |
| High income | 664 (27.1) | 4904 (30.5) |  |  |
| Sleep duration, h, mean (SD) | 7.78 (1.99) | 7.64 (1.59) | <0.001 |  |
| Sleep disturbance |  |  |  |  |
| Not at all | 120 (45.3) | 11214 (61.3) |  |  |
| Several days | 54 (20.4) | 4172 (22.8) |  |  |
| More than half the days | 36 (13.6) | 1323 (7.2) |  |  |
| Nearly every day | 55 (20.8) | 1571 (8.6) |  |  |

Abbreviations: BMI, body mass index; SD, standard deviation.

Table S3. Univariate analysis of the association between depression symptoms with covariates

|  | Statistics | OR (95% CI) | p-value |
| --- | --- | --- | --- |
| Sex |  |  |  |
| Female | 9326 (51.0) | Reference |  |
| Male | 8954 (49.0) | 0.62(0.53,0.72) | <0.001 |
| Age groups |  |  |  |
| ≥18, <40 | 5448 (31.3) | Reference |  |
| ≥40, <60 | 5696 (32.7) | 0.93(0.79,1.09) | 0.360 |
| ≥60 | 6261 (36.0) | 0.84(0.68,1.03) | 0.090 |
| Race/ethnicity |  |  |  |
| Non-Hispanic White | 6379 (34.9) | Reference |  |
| Non-Hispanic Black | 4370 (23.9) | 1.03(0.87,1.22) | 0.740 |
| Mexican American | 2595 (14.2) | 0.88(0.66,1.19) | 0.400 |
| Other Hispanic | 1940 (10.6) | 1.26(1.01,1.56) | 0.040 |
| Other race/multiple races | 2996 (16.4) | 1.07(0.84,1.38) | 0.580 |
| Educational level |  |  |  |
| <High school | 3514 (19.7) |  |  |
| Completed high school | 4316 (24.1) | 0.74(0.60,0.91) | 0.010 |
| >High school | 10051 (56.2) | 0.53(0.45,0.64) | <0.001 |
| Marital status |  |  |  |
| Never married/Widowed /Divorced/Separated | 7120 (40.9) | Reference |  |
| Married/Living with partner | 10275 (59.1) | 0.48(0.41,0.56) | <0.001 |
| Drinking status |  |  |  |
| Never drinking | 2321 (14.8) | Reference |  |
| Former drinker | 817 (5.2) | 2.33(1.49,3.63) | <0.001 |
| Current light/moderate drinker | 9205 (58.7) | 1.44(1.08,1.91) | 0.010 |
| Current heavier drinker | 3348 (21.3) | 2.17(1.66,2.82) | <0.001 |
| Smoking status |  |  |  |
| Never smoker | 9490 (55.9) | Reference |  |
| Former smoker | 4204 (24.8) | 1.39(1.14,1.69) | <0.001 |
| Current smoker | 3268 (19.3) | 3.28(2.78,3.88) | <0.001 |
| Physical activity |  |  |  |
| Inactive | 9353 (51.2) |  |  |
| Moderate | 4233 (23.2) | 0.64(0.51,0.81) | <0.001 |
| Vigorous | 1410 (7.7) | 0.33(0.20,0.52) | <0.001 |
| Both moderate and vigorous | 3284 (18.0) | 0.40(0.32,0.51) | <0.001 |
| BMI category |  |  |  |
| <18.5 kg/m2 | 286 (1.6) | Reference |  |
| 18.5 to <25.0 kg/m^2^ | 4568 (25.3) | 0.48(0.25,0.91) | 0.030 |
| 25.0 to <30.0 kg/m^2^ | 5700 (31.5) | 0.37(0.19,0.70) | 0.003 |
| ≥30.0 kg/m^2^ | 7522 (41.6) | 0.63(0.36,1.11) | 0.100 |
| Family income |  |  |  |
| Low income | 4801 (29.8) | Reference |  |
| Medium income | 6391 (39.7) | 0.53(0.45,0.61) | <0.001 |
| High income | 4904 (30.5) | 0.27(0.23,0.33) | <0.001 |
| Comorbid condition |  |  |  |
| No | 9843 (53.9) | Reference | <0.001 |
| Yes | 8417 (46.1) | 1.88(1.72,2.04) |  |
| Sleep duration |  |  | <0.001 |
| <6 h | 1767 (9.7) | Reference |  |
| 6 to 8 h | 10917 (59.7) | 0.39(0.31,0.47) | <0.001 |
| >8 h | 5595 (30.6) | 0.62(0.49,0.78) | <0.001 |
| Sleep disturbance |  |  |  |
| Not at all | 11214 (61.3) | Reference | <0.001 |
| Several days | 4172 (22.8) | 5.78(4.12, 8.12) | <0.001 |
| More than half the days | 1323 (7.2) | 30.52(20.04, 46.47) | <0.001 |
| Nearly every day | 1571 (8.6) | 95.21(72.84,124.45) | <0.001 |

Abbreviations: BMI, body mass index; CI, confidence interval; OR, odds ratio

Table S4. Associations of sleep midpoint with depression symptoms after adjusting for covariates that used the dummy variables to handle missing values (n = 18279).

|  | OR (95% CI) | p-value |
| --- | --- | --- |
| One-line model | | |
| per 1 hour increase | 1.02 (1.00, 1.04) | 0.021 |
| Quantile regression model | | |
| Group 1 (>6:30 AM to ≤1:30 AM the next day) | 0.92 (0.69, 1.22) | 0.547 |
| Group 2 (>1:30 AM to ≤2:30 AM) | Reference (1) |  |
| Group 3 (>2:30 AM to <3:25 AM) | 0.83 (0.53, 1.30) | 0.381 |
| Group 4 (≥3:25 AM to ≤6:30 AM) | 1.23 (1.00, 1.52) | 0.053 |
| Piecewise regression model | | |
| Sleep midpoint after 6:30 AM and before 2:30 AM the next day | 1.01 (0.99, 1.03) | 0.264 |
| Sleep midpoint from 2:30 AM to 6:30 AM | 1.19 (1.07, 1.33) | 0.003 |
| P value for nonlinear | <0.001 |  |

Abbreviations: CI, confidence interval; OR, odds ratio; Q, quantile; RCS, restricted cubic spline

Covariates that showed a nonlinear relationship with the outcome in the univariate analysis were adjusted as RCSs.

Adjust for age, sex, race/ethnicity, education level, marital status, family income, body mass index (RCS), smoking status, drinking status, physical activity, comorbid condition, sleep duration (RCS), and sleep disturbance. Sleep duration was a continuous variable and only one sample had missing values. The dummy variable method could not be used to handle missing values, so this sample was deleted.


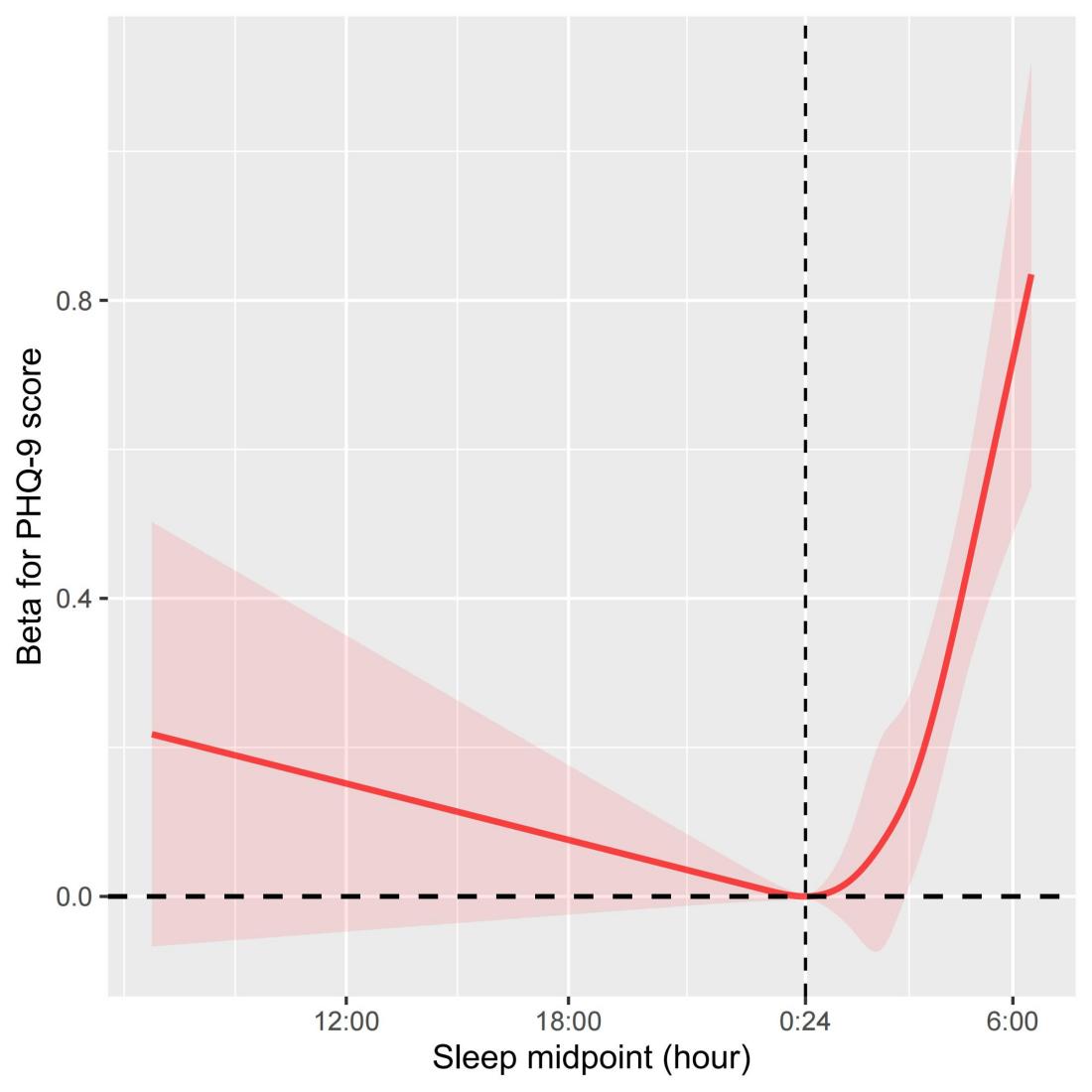


Fig. S2. Weighted restricted cubic spline of the association between sleep midpoint and Patient Health Questionnaire-9 score (with the sleep subitem removed). Shaded areas are 95% confidence intervals. The model is adjusted for age, sex, race/ethnicity, education level, marital status and family income, body mass index, smoking status, drinking status, physical activity, comorbid condition, sleep duration, and sleep disturbance (Model 3).

Table S5. Threshold effect analysis of sleep midpoint on Patient Health Questionnaire-9 score (n = 18280)

| Crude model^a^ | | | Adjusted model^b^ | | |
| --- | --- | --- | --- | --- | --- |
| Inflection point | β (95% CI) | p-value | Inflection point | β (95% CI) | p-value |
| Sleep midpoint after 6:30 AM and before 2:18 AM the next day | -0.09 (-0.11, -0.06) | <0.001 | Sleep midpoint after 6:30 AM and before 0:24 AM the next day | 0.01 (-0.05, 0.06) | 0.791 |
| Sleep midpoint from 2:18 AM to 6:30 AM | 0.61 (0.50, 0.72) | <0.001 | Sleep midpoint from 0:24 AM to 6:30 AM | 0.11 (0.05, 0.17) | 0.001 |
| P value for nonlinear | <0.001 |  | P value for nonlinear | <0.001 |  |

Abbreviations: CI, confidence interval; RCS, restricted cubic spline.

Covariates that showed a nonlinear relationship with the outcome in the univariate analysis were adjusted as RCSs.

Adjust for age, sex, race/ethnicity, education level, marital status, family income, body mass index (RCS), smoking status, drinking status, physical activity, comorbid condition, sleep duration (RCS), and sleep disturbance (Model 3).
